# Macular pigment response to a supplement containing meso-zeaxanthin, lutein and zeaxanthin

**DOI:** 10.1186/1743-7075-4-12

**Published:** 2007-05-11

**Authors:** Richard A Bone, John T Landrum, Yisi Cao, Alan N Howard, Francesca Alvarez-Calderon

**Affiliations:** 1 Department of Physics, Florida International University, 11200 SW 8th Street, Miami, FL 33199, USA; 2 Department of Chemistry and Biochemistry, Florida International University, 11200 SW 8th Street, Miami, FL 33199, USA; 3 Downing College, University of Cambridge, Cambridge CB2 1DQ, UK

## Abstract

**Background:**

Age-related macular degeneration (AMD) is a disease with multiple risk factors, many of which appear to involve oxidative stress. Macular pigment, with its antioxidant and light-screening properties, is thought to be protective against AMD. A result has been the appearance of dietary supplements containing the macular carotenoids, lutein and zeaxanthin. More recently, a supplement has been marketed containing, in addition, the third major carotenoid of the macular pigment, meso-zeaxanthin. The purpose of the study was to determine the effectiveness of such a supplement in raising macular pigment density in human subjects.

**Methods:**

A 120 day supplementation study was conducted in which 10 subjects were given gel-caps that provided 20 mg/day of predominantly meso-zeaxanthin, with smaller amounts of lutein and zeaxanthin. A second group of 9 subjects were given gel caps containing a placebo for the same 120 day period. Prior to and during the supplementation period, blood serum samples were analyzed by high performance liquid chromatography for carotenoid content. Similarly, macular pigment optical density was measured by heterochromatic flicker photometry. Differences in response between the supplementation and placebo groups were tested for significance using a student's t-test.

**Results:**

During supplementation with the carotenoids, blood samples revealed the presence of all three carotenoids. Macular pigment optical density, measured at 460 nm, rose at an average rate of 0.59 ± 0.79 milli-absorbance unit/day in the 10 supplemented subjects. This was significantly different from the placebo group (9 subjects) for whom the average rate was -0.17 ± 0.42 milli-absorbance units/day.

**Conclusion:**

We have shown for the first time that meso-zeaxanthin is absorbed into the serum following ingestion. The data indicate that a supplement containing predominantly meso-zeaxanthin is generally effective at raising macular pigment density, and may turn out to be a useful addition to the defenses against AMD.

## Background

Macular pigment (MP), first identified in the mid-1980s as a combination of lutein and zeaxanthin [1-3], was subsequently shown to be characterized by the presence of specific stereoisomers of these two carotenoids [4]. While lutein (L) is present as a single stereoisomer, [(3R,3'R,6'R)-β, ε-carotene-3,3'-diol], zeaxanthin occurs primarily as a mixture of [(3R,3'R)-β, β-carotene-3,3'-diol] and [(3R,3'S)-β, β-carotene-3,3'-diol], with a much smaller amount of [(3S,3'S)-β, β-carotene-3,3'-diol]. The first two predominant zeaxanthin isomers are referred to as zeaxanthin (Z) and *meso*-zeaxanthin (MZ), respectively. Of these carotenoids, only L and Z are normally consumed in a roughly seven-to-one ratio [5] whereas MZ is an unusual and consequently rare isomer in the diet. MZ is present in significant quantities in commercially produced chickens and eggs in Mexico where it is commonly added to the feed to achieve desirable coloration in these products. Approximately 3000 kg of MZ are sold each year for this purpose [6]. In the US population, L and Z are abundant in the serum, but MZ cannot normally be detected in the serum. This observation led to a hypothesis that MZ is formed in the retina as a conversion product of L [4,7]. In vitro, the conversion of L into MZ is readily achieved in a base-catalyzed reaction [4], and this is the basis of an industrial process for its synthesis and use in poultry feed. The conversion hypothesis is supported by the distribution of the individual carotenoids in the retina. In the central 10°, there is more MZ and less L relative to Z, whereas in the periphery (>35° eccentricity) the situation is reversed [8]. This suggests that the postulated conversion process operates with greater efficiency in the foveal center compared with the peripheral retina. Monkeys raised on a carotenoid-free diet, and then supplemented with L only, subsequently exhibited both L and MZ in the MP. Those supplemented with Z only exhibited no MZ in their MP, only Z. These data are strong evidence in support of the L-to-MZ conversion hypothesis [9].

**Figure 1:**
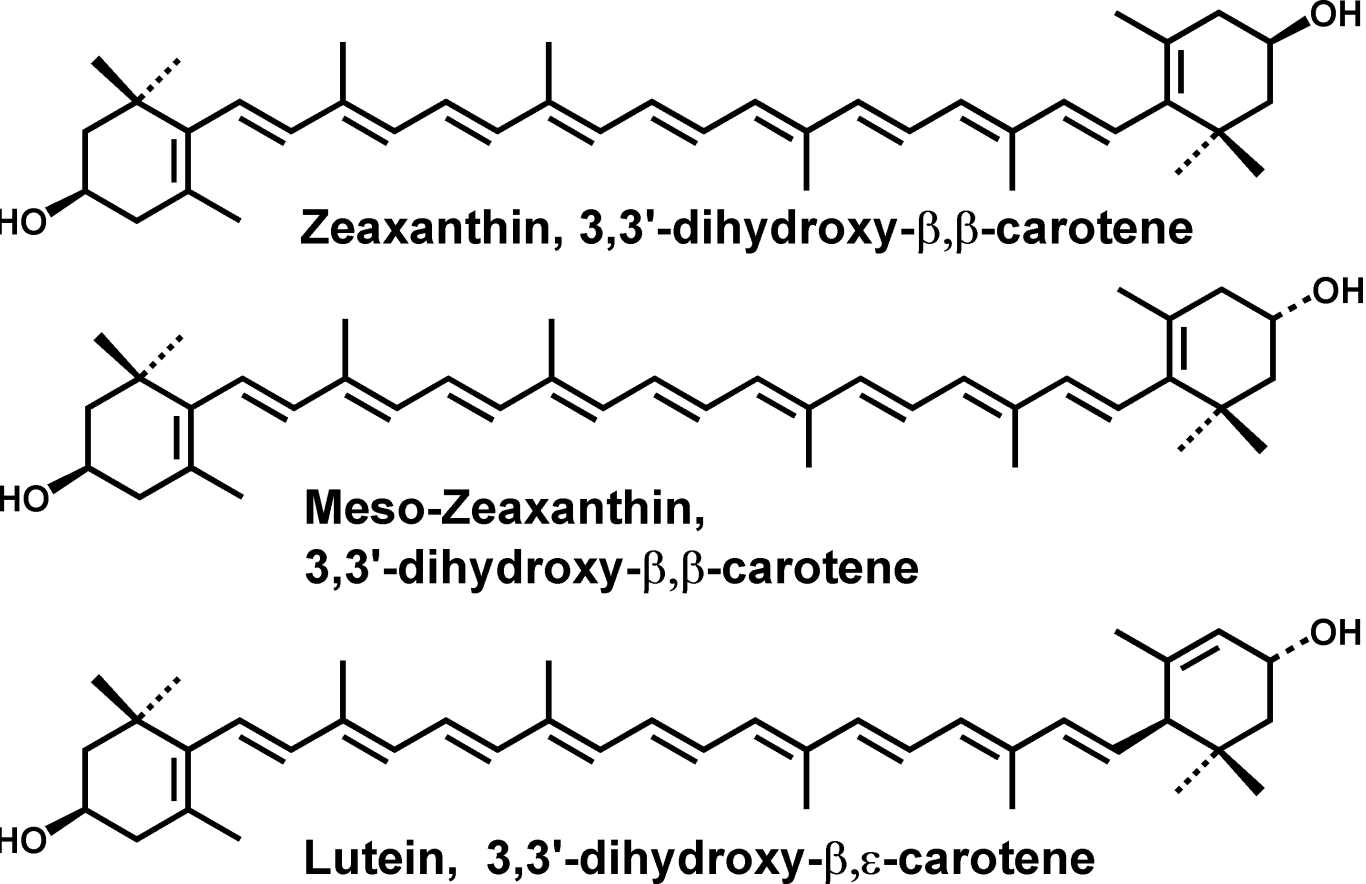
Structures of the major constituents of the macular pigment.

The absolute configuration of the hydroxyl groups located on the 3 and 3' carbon atoms of the carotenoid end-groups is identical in the L and MZ molecules. Thus the conversion of L into MZ need only involve a shift of one carbon-carbon double bond in the ε-ring of L, thereby increasing the conjugation (see Fig. 1). An alternative mechanism for the formation of MZ posits that the metabolite, dehydrolutein, gives rise to MZ through an enzymatic reduction pathway [10]. Indeed the keto-carotenoid canthaxanthin does undergo reduction in the human and primate retina lending credence to this possibility [11]. However, in plasma, dehydrolutein is formed from both L [12] and Z [13]. If these processes also occur in the eye, and MZ is formed from this metabolite, it should have been found in Z- as well as L-supplemented monkeys. Whatever pathway is involved, it would probably involve enzymatic control in order to be consistent with the observation that the proportion of MZ within the retina is dependent upon the location.

At this stage we can speculate on the possible advantages that such a conversion might afford to the eye. It has been proposed that MP protects the macula by two processes [14]. Its presence in the photoreceptor axons [15], together with the range of spectral absorbance (~400 – 500 nm) provides MP with the ability to shield posterior tissues, such as the photoreceptor outer segments and the RPE, from actinic blue light. Additionally, its presence in the outer segments and RPE [16,17] may mitigate the photooxidative damage caused by blue light [18] via its antioxidant and free-radical scavenging properties. In vitro experiments indicate that Z is a more potent antioxidant than L [18]. In one study, quenching of singlet oxygen by Z was approximately twice as effective as quenching by L [19]. The reason is presumably due, at least in part, to the extended conjugation of Z compared with L. MZ shares this electronic feature with Z and therefore should possess the same antioxidant potential as Z. It has also been reported that in association with a zeaxanthin binding protein, the pi isoform of glutathione S-transferase, MZ provides slightly better protection against lipid membrane oxidation than Z [20]. Without the binding protein, the situation is reversed. Thus it may be advantageous to the macula to increase the ratio of total zeaxanthin (Z+MZ) to L. In the serum the ratio of Z to L is about 1:4. A secondary advantage may be the modest but significant shift to longer wavelengths of the Z chromophore relative to that of L resulting in an increase in the wavelength range of blue light screening compared with that which would be provided if the MP was predominantly composed of L. A third advantage may arise from the actual placement of the carotenoid molecules. Observations indicate that L and Z may be present within cellular membranes [21,22]. Z appears to span the membrane in a perpendicular orientation whereas L tends to lie close to the membrane surface [23]. These configurations place Z's protective oxidation sites closer to the readily oxidizable polyunsaturated fatty acid chains in the interior of the membrane.

The purpose of the study was to investigate the potential of ingested MZ in combination with L and Z to increase the optical density of MP. The study was motivated by the recent emergence of dietary supplements containing significant amounts of MZ in addition to L and Z.

## Methods

### Subjects

Eight male and 2 female subjects were recruited from the University community for the supplementation study. Their ages ranged from 21 to 58 years (mean 30.5 ± 10.9 years). Two more subjects were recruited for a more detailed analysis of blood serum carotenoids. Their ages were 51 and 61. Subsequently, a comparison group of subjects was recruited to receive a placebo (placebos only became available after the study had been initiated). The group consisted of 5 males and 4 females with an age range of 19 to 31 years (mean 22.1 ± 3.6 years). While our study did not conform to the standard, double blind, placebo-controlled trial in which subjects are randomly assigned to the treatment and placebo groups, it did offer the opportunity of distinguishing MP changes due to supplementation from those that might occur on a more random basis. It should also be noted that the study took place in South Florida where the diet tends not to be seasonal owing to the constant availability of commonly consumed fruits and vegetables. Therefore we believe that not running the supplementation and placebo groups concurrently would not introduce a bias into the results. Furthermore, while the difference in the mean ages of the groups was just significant (p < 0.05), we are not aware of any age-related differences in the ability of individuals to modify their MP by supplementation.

Exclusion criteria for potential subjects were 1) being a smoker or having smoked in the previous 12 months, 2) any visual pathology, 3) inability during practice sessions to provide acceptable MP optical density (MPOD) measurements, and 4) the use of oral supplements containing significant (>0.25 mg/day) amounts of L and/or Z. Apart from the latter restriction, subjects were free to follow their normal diet. Subjects signed an informed consent form in accordance with the requirements of the Institutional Review Board, and the study conformed to the tenets of the Declaration of Helsinki. Each subject was given training in heterochromatic flicker photometry, the method used to determine MPOD. Official enrollment in the study followed a practice period during which the subjects were required to achieve acceptable MPOD measurements (standard error in the mean based on a set of 5 central and 5 peripheral settings ≤ 0.020 absorbance units. See below.).

### Supplementation

Subjects in the supplementation group were given supplies of gel caps containing unesterified carotenoids: 14.9 mg of MZ, 5.5 mg of L, and 1.4 mg of Z as a suspension in soybean oil, and were informed of the contents. The composition of the gel caps was determined by HPLC (see below). The carotenoid mixture was produced by Industrial Orgánica SA (Monterrey, Mexico) in a base-catalyzed reaction of carotenoids (~93% L, 7% Z) extracted from marigolds (Targetes sp.). Safety of MZ has been verified in a recent toxicity trial. Based on the trial, the no-observed-adverse-effect-level (NOAEL) of MZ in rats was >200 mg/kg/day [24]. The product has also been tested for mutagenicity, using the Ames test, with negative results [25].

Subjects in the placebo group were given gel caps containing only a small quantity of vegetable oil. Unlike those in the supplementation group, the placebo group was blinded, being informed that the gel caps contained either carotenoids or a placebo. Subjects in both groups were instructed to take the gel caps on a daily basis, with a meal, for a period of 120 days.

### MPOD measurements

MPOD was determined for each subject by heterochromatic flicker photometry (HFP) [26]. The subjects viewed a 1.5° stimulus that alternated between 460 and 540 nm, and adjusted the intensity of the former until flicker was minimized. Five repeat adjustments were made while viewing the stimulus centrally (frequency 30 Hz), and 5 repeat reference adjustments were made while viewing the stimulus peripherally at 8° eccentricity (frequency 22 Hz). After each setting, the wedge filter controlling the 460 nm intensity was automatically given a random offset. The log ratio of the averages of the two sets of intensity measurements is equal to the difference in MPOD at 460 nm between the fovea and the peripheral reference site. Assuming MPOD is negligible at the peripheral site, the procedure essentially provides the average MPOD within the central 1.5° [27]. However, this may not be the case for subjects receiving high doses of carotenoids. Rodriguez-Carmona et al. found that after supplementation with 20 mg per day of L and/or Z, the slope of the MPOD distribution remained finite even as far out as 8° eccentricity [28] implying that MPOD could not be assumed to be zero at this location. Thus the central MPOD may be slightly underestimated by HFP at the end of a supplementation study, even if the procedure employs a reference site as far out as 8°. Also, in the parafovea (5.5° eccentricity), MPOD may, according to one study [29], increase with age and this, for the same reason, could cause an apparent decrease in central MPOD with age when using a 5.5° or, conceivably, an 8°, reference site.

MPOD measurements were made at least 4 times prior to supplementation, then twice weekly during the 120 day supplementation period and for the 4 week period following supplementation. (Allowances were made for holidays, etc., resulting in occasional gaps in the data.) It should be noted that these measurements represent a self-administered test with no intervention by the operator. The subjects record each wedge setting by pressing a push-button and a microprocessor automatically calculates their MPOD and associated standard error in the mean. This effectively eliminates any operator or subject bias that might otherwise differentially influence the results of either the MZ or placebo groups.

### Serum analysis of carotenoids

Serum samples were obtained from each subject in order to monitor changes in L and Z concentration. Analysis was conducted by an assistant who was blinded with regard to the origin of the serum samples (MZ or placebo). Two samples were obtained prior to supplementation to establish a baseline. During supplementation, serum samples were obtained every 2 weeks. Carotenoids were extracted from serum by methods that have been described previously [30], and included the addition, prior to extraction, of a known amount of an internal standard (monopentyl lutein ether) to each milliliter of serum. HPLC of the extracts was conducted on a reversed-phase system using a 250 × 4.6 mm Ultracarb ODS 3 μm column (Phenomenex, Torrance CA). The mobile phase was acetonitrile/methanol (85:15) at 1 mL/min with 0.1% triethylamine added to inhibit degradation of carotenoids during elution.

This system is capable of producing baseline separation of L and the combined zeaxanthin stereoisomers, but does not separate Z from MZ. In order to determine whether MZ was absorbed into the serum along with L and Z, serum samples were obtained from 2 additional subjects, A and B, prior to supplementation and after 6 weeks of supplementation. Carotenoids extracted from these samples were analyzed by HPLC on a 250 × 4.6 mm Chiralpak AD column consisting of a silica support derivatized with a chiral polysacharide (Daicel Chemical Industries, Osaka, Japan) and a using a mobile phase consisting of hexane and isopropyl alcohol. Elution was carried out at a flow rate of 0.8 mL/min starting at 90% hexane and 10% isopropyl alcohol and increasing to 100% hexane over a 55 min gradient. Stereoisomers were identified by comparison with the elution order of known standards.

The same systems were used for the analysis of carotenoids in the supplement.

## Results

The presence in the serum of MZ, in addition to L and Z, resulting from supplementation was confirmed in the two subjects, A and B, who participated in this part of the study. Prior to supplementation, the serum concentrations of L, Z, and MZ were 22.9, 4.4, and 0.0 nmol/dL, and 14.8, 2.1, and 0.0 nmol/dL, for subjects A and B respectively. After 6 weeks of supplementation, these values were 23.9, 8.7, and 4.4 nmol/dL for subject A, and 45.4, 15.4, and 14.5 nmol/dL for subject B.

For the other 10 subjects in the supplementation study (subject #s 1–10), we determined the increases in L and combined Z + MZ in the serum by averaging each subject's pre-supplementation values as well as the values obtained from week 6 to the end of the supplementation period. The latter average was not affected by the increase in carotenoid concentration in the serum that normally occurs during the early phase of supplementation [31]. The results are shown in Table 1. Prior to supplementation, the average concentrations for the 10 subjects were 30.5 ± 12.5 (range 16.4 to 54.2) nmol/dL for L, and 9.7 ± 4.8 (range 3.6 to 19.0) nmol/dL for Z + MZ. During supplementation (i.e. from week 6 to the end of supplementation), these values rose to 38.0 ± 12.0 (range 24.2 to 62.3) nmol/dL for L, and 26.4 ± 6.5 (range 13.6 to 35.0) nmol/dL for Z + MZ.

**Table 1:** Serum responses for 10 subjects in supplementation group. Baseline and plateau values refer to averages obtained prior to supplementation and from week 6 to the end of supplementation, respectively.

Subject Number	Baseline L concentration ± SD (nmol/dL)	Plateau L concentration ± SD (nmol/dL)	Baseline Z+MZ concentration ± SD (nmol/dL)	Plateau Z+MZ concentration ± SD (nmol/dL)
1	36.9 ± 17.8	42.4 ± 6.7	5.6 ± 0.4	25.1 ± 5.2
2	16.4 ± 1.9	34.1 ± 12.3	7.4 ± 1.6	34.9 ± 15.4
3	22.6 ± 3.5	26.3 ± 2.7	8.7 ± 1.1	22.9 ± 3.7
4	35.8 ± 1.8	36.4 ± 6.9	14.1 ± 5.6	29.6 ± 8.8
5	18.2 ± 1.4	25.5 ± 5.8	6.1 ± 0.4	22.1 ± 5.7
6	42.7 ± 0.9	62.3 ± 18.1	19.0 ± 3.0	35.0 ± 9.3
7	16.7 ± 3.3	24.2 ± 4.9	7.3 ± 0.9	13.6 ± 3.5
8	54.2 ± 5.2	45.2 ± 10.6	11.0 ± 4.0	30.4 ± 0.8
9	34.0 ± 6.8	49.1 ± 4.2	14.0 ± 3.8	27.0 ± 1.2
10	27.1 ± 3.8	34.6 ± 1.2	3.6 ± 2.6	23.4 ± 0.6
				
Mean ± SD	30.5 ± 12.5	38.0 ± 12.0	9.7 ± 4.8	26.4 ± 6.5

For the placebo group (subject #s 11–19), the average serum L of the subjects changed from 19.5 ± 6.4 (range 4.6 to 27.9) prior to taking the placebo to 30.7 ± 22.8 (range 6.4 to 79.1) nmol/dL from week 6 to the end of "supplementation." For Z, the value changed from 7.6 ± 3.4 (range 2.1 to 10.0) to 15.8 ± 15.3 (range 2.9 to 50.3) nmol/dL. Neither of these average changes, which may have been due to dietary changes, was significant according to a 2-sided t-test (p = 0.14 for L, p = 0.13 for Z), nor was any individual change significant.

**Figure 2:**
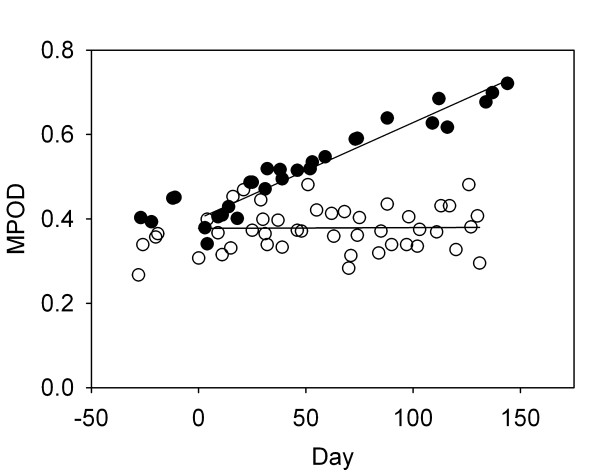
Macular pigment optical density (MPOD) response to supplementation in the left eye of subject # 1 (filled circles) and the right eye of subject # 6 (open circles). Supplementation began on day zero.

MPOD response in each subject was quantified by calculating the average rate of change in MPOD, measured in milliabsorbance units per day (mAU/day), and the associated standard error (SE) for each eye of each subject during the study period. Data for the left eye of subject # 1 (supplementation group), who had a particularly robust response and little scatter in the MPOD measurements, and for the right eye of subject # 6 (supplementation group), who had no significant change in MPOD combined with a lot of scatter, are shown in Fig. 2. The short-term, session-to-session variability is typical of data obtained by HFP, and is probably a measurement artifact. The rate of change in MPOD for each eye of each subject was calculated using all MPOD measurements for that eye from day zero (start of supplementation) onwards. Based on the observation that some subjects (e.g. subjects 2, 4, 9) had significantly different initial MPODs in their left and right eyes, we have treated the responses in each eye of a subject as independent observations. The complete results for the supplementation group are presented in Table 2. The table includes p-values, based on a 2-sided t-test, that indicate whether the measured rates of change in MPOD were significantly different from zero. Significant rates of increase in MPOD were observed in 12 eyes, no significant changes occurred in 7 eyes, and, surprisingly, a significant decrease occurred in one eye.

**Table 2:** Macular pigment optical density (MPOD) response to supplementation. MPOD data for the supplementation group obtained prior to supplementation together with the rates of change in MPOD during supplementation. The p-values indicate whether these rates are significantly different from zero. (AU = absorbance unit)

Subject/eye	MPOD pre AU	SD pre AU	Rate of change in MPOD ± SE, mAU/day	p	Significance of rate of change
1/L	0.421	0.016	2.22 ± 0.11	< 0.0001	Signif. increase
1/R	0.446	0.025	2.01 ± 0.06	< 0.0001	Signif. increase
2/L	0.779	0.015	-0.18 ± 0.30	0.57	No signif. change
2/R	0.679	0.016	0.17 ± 0.38	0.66	No signif. change
3/L	0.286	0.018	0.27 ± 0.10	0.012	Signif. increase
3/R	0.306	0.011	-0.04 ± 0.09	0.69	No signif. change
4/L	0.468	0.019	1.23 ± 0.21	< 0.0001	Signif. increase
4/R	0.355	0.022	1.58 ± 0.28	< 0.0001	Signif. increase
5/L	0.254	0.018	0.51 ± 0.14	0.0009	Signif. increase
5/R	0.265	0.027	-0.46 ± 0.18	0.016	Signif. decrease
6/L	0.351	0.019	-0.22 ± 0.16	0.18	No signif. change
6/R	0.362	0.014	0.02 ± 0.21	0.94	No signif. change
7/L	0.335	0.017	0.92 ± 0.34	0.015	Signif. increase
7/R	0.304	0.02	-0.57 ± 0.35	0.12	No signif. change
8/L	0.718	0.013	0.65 ± 0.25	0.016	Signif. increase
8/R	0.722	0.014	0.44 ± 0.32	0.17	No signif. change
9/L	0.344	0.012	1.07 ± 0.29	0.0009	Signif. increase
9/R	0.421	0.011	1.33 ± 0.35	0.0008	Signif. increase
10/L	0.96	0.008	0.43 ± 0.05	< 0.0001	Signif. increase
10/R	0.854	0.008	0.41 ± 0.04	< 0.0001	Signif. increase

MPOD results for the placebo group are shown in the same format in Table 3. For one subject, there was a significant decrease in MPOD in both eyes, and for another subject, there was a significant decrease in one eye only. No significant changes in MPOD were observed in any other subject's eye in the placebo group.

**Table 3:** Macular pigment optical density (MPOD) response to placebo. See Table 2 for details.

Subject/eye	MPOD pre AU	SD pre AU	Rate of change in MPOD ± SE, mAU/day	p	Significance of rate of change
11/L	0.244	0.034	0.01 ± 0.43	0.99	No signif. change
11/R	0.246	0.055	-0.20 ± 0.26	0.46	No signif. change
12/L	0.570	0.043	-0.99 ± 0.23	0.0001	Signif. decrease
12/R	0.551	0.105	-0.10 ± 0.22	0.65	No signif. change
13/L	0.473	0.065	0.00 ± 0.30	0.99	No signif. change
13/R	0.397	0.077	0.66 ± 0.41	0.12	No signif. change
14/L	0.825	0.067	0.03 ± 0.94	0.98	No signif. change
14/R	0.922	0.186	0.36 ± 0.33	0.30	No signif. change
15/L	0.328	0.050	0.14 ± 0.22	0.53	No signif. change
15/R	0.334	0.058	-0.11 ± 0.24	0.66	No signif. change
16/L	0.492	0.048	-0.72 ± 0.16	<0.0001	Signif. decrease
16/R	0.527	0.022	-0.60 ± 0.18	0.0020	Signif. decrease
17/L	0.579	0.036	-0.64 ± 0.87	0.48	No signif. change
17/R	0.482	0.054	-0.32 ± 0.49	0.52	No signif. change
18/L	0.628	0.041	-0.28 ± 0.30	0.36	No signif. change
18/R	0.625	0.057	0.43 ± 0.28	0.13	No signif. change
19/L	0.739	0.143	-0.44 ± 0.33	0.19	No signif. change
19/R	0.693	0.076	-0.30 ± 0.36	0.41	No signif. change

## Discussion

Data from subjects A and B indicate for the first time that MZ, while not a significant component of a normal diet, is nonetheless absorbed into the serum. The subjects were not on controlled diets and this could account, at least in part, for the very different responses that were observed. The L, Z and MZ in the supplement were in the ratio 1.0:0.3:2.7 while, in the serum, the increases in these carotenoids resulting from supplementation were in the ratio 1.0:4.3:4.4 for subject A, and 1.0:0.43:0.47 for subject B. Thus the serum responses for these two subjects revealed no common pattern, and the relative efficiencies with which L, Z and MZ are absorbed into the serum cannot be proposed without the benefit a larger study involving more subjects and a detailed knowledge of their individual diets. Data from the reversed-phase HPLC analysis of serum L and combined Z + MZ in the other 10 subjects in the supplementation group were reasonably consistent with expectations based on the composition of the supplement. In the supplement, the L:Z + MZ ratio was 1.0:3.0. In the serum, averaged for all 10 subjects, the increases in L and Z + MZ resulting from supplementation were in the ratio1.0:2.2. There was a slight trend towards larger increases in L and Z+MZ in subjects whose pre-supplementation levels of these carotenoids were lower, but not at the level of statistical significance.

The magnitudes of the average increases in L and Z+MZ in the supplemented subjects were quite modest in comparison with those reported in subjects supplemented with comparable amounts of L only or Z only. For example, an average ~3-fold increase in serum L was reported in subjects taking 5 mg of L per day for 6 months [32]. In another 6 month study, subjects received a daily dose of either 10 mg of L, 10 mg of Z, or 10 mg of L combined with 10 mg of Z [33]. The average serum L increase was ~7-fold for the L group, the average serum Z increase was ~27-fold for the Z group, and the average serum L and Z increases were ~4-fold and 14-fold respectively for the group receiving both L and Z. A likely factor influencing the bioavailability of the carotenoid is the formulation of the supplement. There is also some evidence for competition among carotenoids for uptake into the serum when administered in combination [34]. Both of these factors may have been influential in the present study.

Consistent with other L and Z supplementation studies [30,31], MP responses were varied in terms of the increases in MPOD among the 10 subjects in the supplementation group. Four subjects had significant rates of increase in MPOD in both eyes, and 4 others had significant rates of increase in one eye only. The average rate ± SD for the group was 0.59 ± 0.79 mAU/day. For the placebo group, there were no significant rates of increase, and the average ± SD was -0.17 ± 0.42 mAU/day. According to a 2-sided t-test, the averages for the 2 groups differed significantly (p < 0.002). The results obtained for the supplementation group may be compared with our earlier studies in which we reported results averaging 1.13 ± 0.10 mAU/day for a 30 mg/day L study using 2 subjects [31] and 0.48 ± 0.16 mAU/day for a 30 mg/day Z study, again using 2 subjects [30]. On a per milligram basis, the average rate of increase in MPOD obtained in the current study is very similar to that obtained in the L study and approximately double the value obtained in the Z study. However, judgment in making these comparisons should be tentative based on the small numbers of subjects in the earlier studies.

For this small study, we found no correlation between the rate of increase in MPOD in the supplemented subjects and either the plateau concentration (week 6 to the end of supplementation), or the change in concentration, of L or Z+MZ. However, when we restricted our analysis to those subjects who were more skillful in HFP, as judged by the scatter in their data, an interesting trend emerged. For subjects whose rates of change in MPOD were accompanied by a standard error ≤ 0.21 mAU/day, these rates were positively correlated with the increase in serum Z+MZ (R^2 ^= 0.31), though not quite reaching the level of statistical significance (p = 0.078). Therefore it is possible that the MP responses that we observed in our subjects were due in large part to the presence of the major constituent of the supplement, MZ, that was ~10 times as abundant as Z. However, a final conclusion must await testing of a supplement containing MZ only.

Subject #5 in the supplementation group produced an anomalous result consisting of significant rates of increase and decrease in MPOD in the left and right eyes respectively. Assuming that this subject was providing reliable data, the decrease seen in the right eye may have been due to a faster build-up of MP at 8° eccentricity compared with the fovea, as discussed earlier. Significant decreases in MPOD in the placebo group may have been due to a change of diet during the study period.

Data from the current and previous studies are limited by sample size, making it difficult to argue in support of any one of the three carotenoids, L, Z, or MZ, as the preferred means of raising MP density. On the other hand, we know that a significant fraction of L in the central retina is converted to MZ. In the center of the retina, the ratio of MZ to L is highest and approaching 1:1. This observation suggests that the efficiency of conversion of L to MZ is generally below 50%. Furthermore, it is possible that the conversion process may involve a net destruction of carotenoid. As evidence, data from autopsy eyes indicate that the (L + MZ):Z ratio varies throughout the retina from ~2:1 in the center, where most of the conversion is occurring, to ~3:1 in the periphery [7]. Thus there may be an advantage in providing MZ in a supplement at the expense of L if the goal is to raise the overall zeaxanthin level and potentially improve the degree of retinal protection.

## Conclusion

Our study shows that a supplement containing the macular carotenoids, L, Z and MZ, but principally MZ, is effective at raising MPOD in the majority of subjects. Increased MPOD may be an effective means of protecting the aging population from AMD.

## Competing interests

Three of the authors, RAB, JTL and ANH, have a proprietary interest in the use of MZ to raise MPOD and treat macular disorders.

## Authors' contributions

RAB, JTL and ANH conceived the study. ANH provided MZ capsules for the study. RAB designed the flicker photometer, trained subjects in its use and analyzed the data. JTL supervised the blood serum analysis and analyzed the data. YC and FA-C conducted HPLC analysis of blood serum samples. RAB and JTL drafted the manuscript.
